# Bilateral Knee Joint Cooling on Anaerobic Capacity and Wheel Cadence during Sprint Cycling Intervals

**DOI:** 10.3390/healthcare10101951

**Published:** 2022-10-06

**Authors:** Agong Nam, Jihong Park

**Affiliations:** 1 Athletic Training Laboratory, Graduate School of Physical Education, Kyung Hee University, Yongin 17104, Korea; 2 Athletic Training Laboratory, Department of Sports Medicine, Kyung Hee University, Yongin 17104, Korea

**Keywords:** cold application, anaerobic capacity, wheel cadence, muscle temperature

## Abstract

We compared the effect of bilateral knee joint cooling with or without a pre-cooling warm-up on sprint cycling performance to a non-cooling control condition. Seventeen healthy young males (25 ± 2 years, 174 ± 6 cm, 70 ± 9 kg) performed three conditions in a counterbalanced order (condition 1: warming + cooling + cycling; condition 2: cooling + cycling; condition 3: cycling). For warming, a single set of cycling intervals (a 10 s sprint with maximal effort followed by a 180 s active recovery; resistive load 4% and 1% body mass for sprint and recovery, respectively) was performed. For cycling, five sets of cycling intervals were performed. For cooling, 20 min of bilateral focal knee joint cooling was applied. Peak and average values of anaerobic capacity and wheel cadence during each set across conditions were statistically compared. There was no condition effect over set (condition × set) in anaerobic capacity (F_8,224_ < 1.49, *p* > 0.16) and wheel cadence (F_8,224_ < 1.48, *p* > 0.17). Regardless of set (condition effect: F_2,224_ > 8.64, *p* < 0.0002), conditions 1 and 2 produced higher values of anaerobic capacity (*p* ≤ 0.05). Similarly (condition effect: F_2,224_ > 4.62, *p* < 0.02), condition 1 showed higher wheel cadence (*p* < 0.02) than condition 3. A bilateral joint cooling for 20 min with or without pre-cooling warm-up may improve overall sprint cycling capacity during five sets of cycling intervals when compared to the non-cooling condition.

## 1. Introduction

Cold application—extracting heat and energy (reducing temperature)—is an inexpensive and convenient way to achieve clinical benefits such as metabolic reduction [1] and anesthetic effect [2]. In addition to these therapeutic purposes, cooling modalities are applied for enhancing muscle function. For example, cooling palms during a 3 min rest between sets increased the number of repetitions in bench press [3]. Focal knee joint cooling for 20 min increased quadriceps activation and strength [4]. Further, 20 min of bilateral knee joint cooling improved a 20 m sprint [5]. Although not fully understood, possible mechanisms behind this facilitatory effect could at least partially be explained by the decreased recruitment point of the motor unit [6], suppression of the activation of inhibitory neurons [7], and increased firing frequency [8]. Psychologically, changes in sensation at the cooled body part from “tight or heavy” to “loose or light” caused by rapid warm-up could also be involved [9].

Based on the observation of improved sprint ability [5], we could also expect to see a similar effect on riding a cycle ergometer. Assumingly, an improvement in lower-extremity power output and movement efficiency (resulting from the aforementioned neuro-physio-psychological mechanisms) could lead to an improvement in anaerobic capacity and wheel cadence (pedaling rate) during cycling. A previous sprinting improvement [5] was observed approximately 35 min after the knee joint cooling; the 35 min consisted of 5 min of jogging, 20 min of cooling, and 10 min of rapid warming up (five sets of a 2 min intermittent exercise protocol consisting of running and jumping at various intensities). While improved sprinting ability was observed after the activities, the next logical step will be to simplify these series of steps for field application. Particularly, it is important to examine if jogging prior to cooling needs to be performed. If this pre-cooling warm-up is unnecessary, the results would be beneficial for saving time and preserving energy for subsequent exercises. If the pre-cooling warm-up plays a critical role, the results should be tailored to further research.

Therefore, the purpose of this study was to examine the effect of 20 min of bilateral knee joint cooling with or without a pre-cooling warm-up activity on sprint cycling performance. Specifically, we asked: (1) will knee joint cooling result in an improvement in anaerobic capacity and wheel cadence, compared with a non-cooling condition? and (2) if so, will the joint cooling conditions with pre-cooling warm-up show better cycling performance than the joint cooling condition without pre-cooling warm-up? We hypothesized that (1) conditions with knee joint cooling would show better cycling performance than a non-cooling condition, and (2) there would be no difference in cycling performance between the conditions with or without pre-cooling warm-up. The results of this study would directly assist cyclists and coaches by suggesting necessary components for warm-up activities prior to racing. Additional measurements in thigh muscle temperature, fatigue perception, and heart rate during five sets of cycling protocols would also help us explain the cause-and-effect relationship between warm-up activity with knee joint cooling and cycling performance.

## 2. Materials and Methods

### 2.1. Subjects

Our sample size was calculated based on the primary dependent measurement, the anaerobic capacity of cycling sprint (watt/kg). In a similar ergometer test, the average values on healthy adults were between 8 and 10 watt/kg [10,11]. To detect a difference of 2.1 watt/kg with a standard deviation of 2.0 watt/kg (an effect size of 1.0 using an alpha of 0.05 and a beta of 0.2), a minimum of 16 subjects was necessary.

Therefore, seventeen healthy young males (age: 25.0 ± 1.8 years, height: 173.6 ± 6.1 cm, mass: 69.8 ± 9.4 kg) participated in this study. Subjects were physically active (participating in at least 150 min of aerobic exercise per week) and free from cold urticaria, lower-extremity or back injury in the last six months, and lower-extremity or spine surgery in their lifetime. Subjects with any known disease or disorder (e.g., arthritis, diabetes, hypertension) were excluded. Prior to participation, each subject read the testing procedures and provided informed consent, approved by the University’s Institutional Review Board (KHGIRB-19-141).

### 2.2. Testing Procedures

To experience three conditions, subjects reported to the laboratory at the same time for three visits, at least two days apart. Subjects were asked to keep their habitual diet and not to participate in any physical activity for 24 h prior to the visits [5]. The room temperature and relative humidity during the data collection period were kept between 24 and 26 °C and 40 and 60%, respectively.

**Figure 1:**
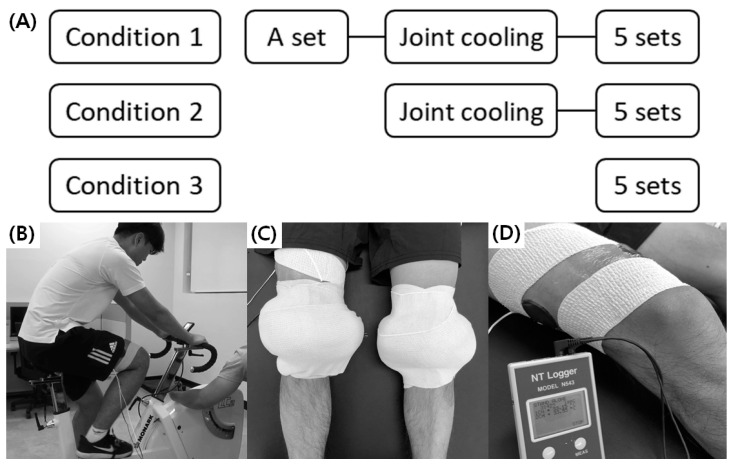
Testing procedures: (**A**) Subjects experienced each condition on three separate visits. (**B**) Each set consisted of a 10 s sprint followed by a 180 s active recovery on a stationary cycling ergometer. (**C**) Two ice bags (directly on the anterior and posterior aspect of the knee joint) were secured with a compression wrap on each side. (**D**) Thermistor probes, covered by neoprene fabric, were attached on the vastus medialis and lateralis.

On the first day of data collection, subjects gave informed consent. Upon arrival to the laboratory for each visit, subjects had a 5 min supine rest on a treatment table. During this resting period, the baseline values of the thigh temperatures, fatigue perception, and heart rate were recorded. Afterward, one of three conditions (condition 1: a set of a cycling protocol + cooling + five sets of a cycling protocol; condition 2: cooling + five sets of a cycling protocol; condition 3: five sets of cycling protocol: Figure 1A) proceeded in a counterbalanced order. The six possible orders (conditions 1–2–3, 2–3–1, 3–1–2, 2–1–3, 3–2–1, or 1–3–2), generated by a random function in a spreadsheet program (Excel, Microsoft Inc., Redmond, WA, USA), were randomly assigned.

For condition 1, a set of a cycling protocol was performed on a stationary cycle ergometer (Monark LC2, Monark Exercise AB, Vansbro, Sweden: Figure 1B) as a pre-cooling warm-up (Figure 1A). A single cycling protocol consisted of a 10 s sprint (resistive load: 4% body mass) followed by a 180 s active recovery interval (resistive load: 1% body mass; 60 rpm). For the sprint cycling, subjects were asked to produce maximal effort to pedal (the self-selected rate of power development), while the frequency of recovery cycling was guided by a metronome. Afterward, subjects received 20 min of bilateral knee joint cooling in a knee extended seated position. An ice bag (a plastic bag with a size of 25 cm × 46 cm, filled with 500 mL crushed ice) was directly attached to the anterior and posterior aspects of the knee joint (covering the patella, medial and lateral aspects of the knee joint line, and popliteal fossa, not the tendon and muscle structures) and was secured with a compression wrap (Figure 1C) on each side. Subjects then continuously performed five sets of the cycling protocols described above. Verbal encouragement “harder, harder, to the end” was provided during the 10 s sprint cycling.

For condition 2, 20 min bilateral knee joint cooling was applied followed by five sets of the cycling protocol. For condition 3, five sets of the cycling protocol were performed. The self-adjusted saddle and handlebar positions were recorded at the first visit for consistency in the remaining visits.

### 2.3. Outcome Measures

Dependent measurements were sprint cycling performance (average and peak anaerobic capacity and wheel cadence), thigh temperatures (vastus medialis and lateralis), fatigue perception (using a 10 cm visual analog scale), and heart rate (using a heart rate monitor).

To test cycling performance, peak and average capacity and wheel cadence during a 10 s cycling sprint during the last five sets of cycling protocol were compared across conditions (a single set of a cycling protocol was performed as a warm-up; thus, not analyzed). Using the ergometer’s built-in computer, data were sampled at 50 Hz for anaerobic capacity (watt) and wheel cadence (revolution per min: rpm) and then wirelessly exported into a spreadsheet program using software (Golden Cheetah v3.4, http://www.goldencheetah.org/ accessed in 11 November 2019). Average and peak values during each set of 10 s cycling sprints (except for the first set for condition 1) were compared.

For the thigh muscle temperature, a digital logger thermometer (sampled at 60 Hz; N543, NT logger, NKTC, Tokyo, Japan) using two separate channels of thermistor probes were used (Figure 1D). Each thermistor probe was attached to the skin superficial to the vastus medialis (VM: channel 1) and lateralis (VL: channel 2) and covered by neoprene fabric (thickness: 3 mm; diameter: 5 cm) and self-adhesive film dressing (Tegaderm Film, 3M, St. Paul, MN, USA). This is a valid method to record a 2.2 cm deep inner tissue by blocking the heat transfer underneath the neoprene cover [12,13]. The values at 1 s prior to the 10 s cycling sprint at each set (except for the first set in case of condition 1) were compared. Five seconds prior to the 10 s cycling sprint at each set of the cycling protocol, subjects were asked to mark where their perception of fatigue was on a 10 cm (0 being unfatigued and 10 being fatigued) visual analog scale (VAS) [14] for fatigue perception.

The heart rate monitor (sampled at 1000 Hz; Polar H10, Polar Electro Inc., New York, NY, USA) was wirelessly connected to a mobile phone application (Polar beat: Run & Fitness). Heart rate (bpm) data were then exported into the spreadsheet. The values at the same time points as the thigh temperature samples were compared.

Room temperature and relative humidity were recorded using a wireless environmental data logger (Kestrel Drop D3, Nielsen-Kellerman Co., Boothwyn, PA, USA).

### 2.4. Statistical Analysis

To test the condition effect over set, we performed a 3 (condition) × 5 (set) mixed model ANOVA for anaerobic capacity and wheel cadence; a 3 (condition) × 6 (baseline and each set) mixed model ANOVA (random variable: subject; fixed variables: condition and set) for thigh temperature, fatigue perception, and heart rate. Tukey–Kramer pairwise comparisons were used for post hoc tests (*p* ≤ 0.05 for all tests; SAS 9.4: SAS Institute Inc., Cary, NC, USA). Cohen’s d effect sizes (d) [15] were calculated when statistical significances exist.

## 3. Results

### 3.1. Cycling Performance

**Figure 2:**
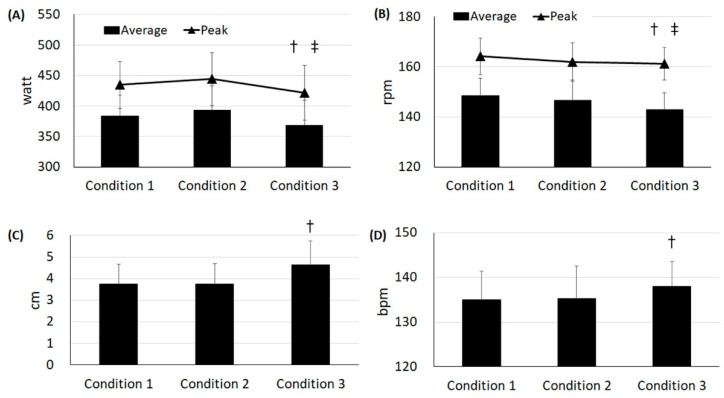
Changes in anaerobic capacity (**A**), cadence (**B**), fatigue perception (**C**), and heart rate (**D**), regardless of set (T bars indicate upper or lower limit of 95% confidence intervals). (**A**) † Average: Condition 3 (369 watt) is different from conditions 1 (383 watt) and 2 (394 watt); ‡ Peak: Condition 3 (422 watt) is different from conditions 1 (444 watt) and 2 (435 watt). (B) † Average cadence: Condition 3 (143 rpm) is different from conditions 1 (148 rpm) and 2 (147 rpm); ‡ Peak cadence: Condition 3 (161 rpm) was different from condition 1 (164 rpm). (C) † Condition 3 (4.6 cm) was different from conditions 1 (3.8 cm) and 2 (3.7 cm). (D) † Condition 3 (138 bpm) was different from conditions 1 (135 bpm) and 2 (135 bpm).

There was no condition effect over set (condition × set) in average (F_8,224_ = 0.72, *p* = 0.67) or peak (F_8,224_ = 1.49, *p* = 0.16: Table 1) values in anaerobic capacity. Regardless of set (condition effect), subjects with knee joint cooling (conditions 1 and 2) showed higher anaerobic capacity than subjects without cooling (average: F_2,224_ = 13.6, *p* < 0.0001; peak: F_2,224_ = 8.64, *p* = 0.0002: Figure 2A). Regardless of condition (set effect), average (F_4,224_ = 4.69, *p* = 0.001) and peak (F_4,224_ = 5.00, *p* = 0.0007) values in anaerobic capacity were reduced at set 4 (*p* = 0.03, d = 0.24) and 5 (*p* = 0.03, d = 0.30), respectively, as compared with those at set 1.

There was no condition effect over set (condition × set) in average cadence (F_8,224_ = 0.41, *p* = 0.91) and peak cadence (F_8,224_ = 1.48, *p* = 0.17: Table 1). Regardless of set (condition effect: F_2,224_ = 10.38, *p* < 0.0001), condition 3 showed 5 (4%, *p* < 0.0001, d = 0.39) and 4 (3%, *p* = 0.008, d = 0.25) fewer wheel rpm as compared with conditions 1 and 2, respectively (Figure 2B). In a comparison of peak cadence (condition effect: F_2,224_ = 4.62, *p* = 0.02), subjects with condition 1 had 3 greater wheel rpm than those with condition 3 (2%, *p* = 0.02, d = 0.20, Figure 2B). Regardless of condition (set effect: F_4,224_ = 3.38, *p* = 0.02), the average cadence between sets 2 and 5 was different (*p* = 0.05, d = 0.28). The highest peak cadence (set effect: F_4,224_ = 11.72, *p* < 0.0001) was recorded at set 2, as compared with sets 1 (*p* = 0.02, d = 0.28), 4 (*p* < 0.0001, d = 0.42), and 5 (*p* < 0.0001, d = 0.53).

### 3.2. Thigh Temperature

**Figure 3:**
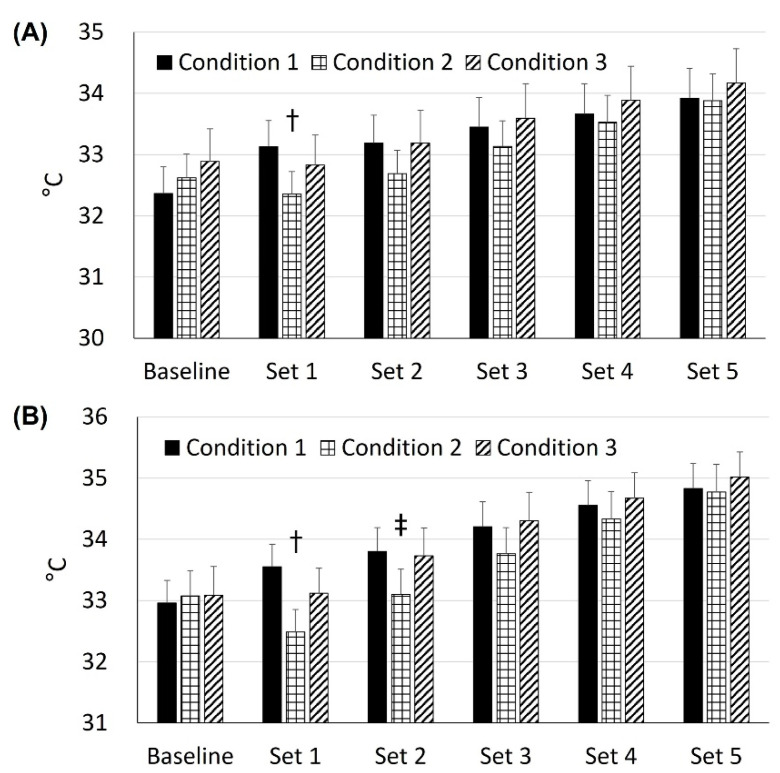
Changes in the temperature on the vastus medialis (**A**) and lateralis (**B**) for each condition over set (T bars indicate upper limit of 95% confidence intervals). (**A**) † Condition 2 (32.4 °C) was lower than condition 1 (33.1 °C) at set 1. (**B**) † Condition 2 (32.5 °C) was different from conditions 1 (33.6 °C) and 3 (33.1 °C) at set 1. ‡ Condition 2 (33.1 °C) was different from conditions 1 (33.8 °C) and 3 (33.7 °C) at set 2.

There was a condition effect over set in the VM temperatures (condition × set: F_10,272_ = 1.94, *p* = 0.04, condition effect: F_2,272_ = 12.61, *p* < 0.0001; set effect: F_5,272_ = 46.33, *p* < 0.0001). The VM temperature under condition 2 was 0.7 °C lower than condition 1′s temperature (32.4 °C vs. 33.1 °C, *p* < 0.01, d = 0.94, Figure 3A) at set 1.

There was also a condition effect over set in the VL temperatures (condition × set: F_10,272_ = 3.34, *p* = 0.0004, condition effect: F_2,272_ = 20.76, *p* < 0.0001; set effect: F_5,272_ = 116.41, *p* < 0.0001). The VL temperature for condition 2 (32.5 °C) was lower than the temperature at conditions 1 (33.6 °C, *p* < 0.0001, d = 1.40) and 3 (33.1 °C, *p* = 0.04, d = 0.79, Figure 3B) at set 1. At set 2, the VL temperature at condition 2 (33.1 °C) was still lower than the temperature at conditions 1 (33.8 °C, *p* = 0.01, d = 0.85) and 3 (33.7 °C, *p* = 0.04, d = 0.70).

### 3.3. Fatigue Perception

There was no condition effect over set in perception of fatigue (condition × set: F_10,272_ = 0.27, *p* = 0.97, Table 2). Regardless of set (condition effect: F_2,272_ = 13.87, *p* < 0.0001), fatigue perception for condition 3 (4.7 cm) was 19% higher than conditions 1 or 2 (3.8 cm for each, *p* < 0.0001, d = 0.43, Figure 2C). Regardless of condition (set effect: F_5,272_ = 47.84, *p* < 0.0001), subjects began to feel fatigue at set 3 (3.9 cm, *p* < 0.0001, d = 0.53) as compared to the baseline (2.8 cm).

### 3.4. Heart Rate

There was no condition effect over set (condition × set) in heart rate (F_10,272_ = 0.57, *p* = 0.84, Table 2). Regardless of set (condition effect: F_2,272_ = 3.19, *p* = 0.04), heart rate for condition 3 (138 bpm) was 2% higher than for conditions 1 or 2 (135 bpm for each, *p* < 0.0001, d = 0.20, Figure 2C). Regardless of condition (set effect: F_5,272_ = 566.45, *p* < 0.0001), subjects’ heart rate immediately increased at set 1 (137 bpm, *p* < 0.0001) and then further increased at sets 3 (149 bpm, *p* < 0.0001, d = 0.90) and 5 (155 bpm, *p* < 0.0001, d = 0.40) from the baseline value (76 bpm).

## 4. Discussion

We were interested in observing how 20 min bilateral knee joint cooling combined with or without a pre-cooling warm-up affects cycling sprint performance during five sets of cycling intervals. Although our results did not show an interaction effect (finding a peak performance at a specific time point under a specific condition), our hypotheses that the conditions with joint cooling, regardless of the pre-cooling warm-up, would show a better cycling sprint performance than a non-cooling condition and that there would be no difference between the cooling conditions were generally accepted. Therefore, the order of cooling and warming up does not seem to be an important factor. We did not observe the peak cycling performance on a specific condition at a specific set during cycling interval protocols. When collapsing data across time points (condition effect), knee joint cooling appears to be beneficial in cycling performance in terms of anaerobic capacity and cadence. Our results, therefore, indirectly support the previous data on physical performance enhancement involved with pre-exercise cooling [3,4,5,16,17]. Less fatigue perception and heart rate in the conditions with knee joint cooling, relative to the non-cooling condition, are also indicative of performance efficiency by pre-exercise cooling.

Statistically, the performance enhancement in cycling sprints was not observed in an interaction (condition × set) but in a condition main effect. Subjects with knee joint cooling (conditions 1 and 2) showed 4 to 5 greater wheel revolutions while completing the five sets of 10 s cycling sprints when compared with those without cooling (condition 3). While the recorded total wheel cadence ranged between 130 and 145 rpm (based on the wheel cadence in each set, which ranged between 26 and 29 rpm) in our study, the results of the current study indicate that subjects who receive knee joint cooling may generate 4 to 5 additional rpm. The wheel circumference of the stationary bike is 2 m (by manufacture), and our results can be further interpreted as being located 8 to 10 m ahead. Although the standardized mean differences in anaerobic capacity and wheel cadence are small (d < 0.39), relative to the previous data on performance enhancement [4,5], this clear advantage of the knee joint cold application should be considered in cycling sprints. Regardless of condition, the cycling performances peaked at set 2 and declined at sets 4 and 5. These results also have practical implications in that the first set of each condition was performed as a warm-up. Along with the data on fatigue perception (fatigued at set 3), the declined cycling performance at sets 4 and 5 could be indicative of general muscular fatigue.

While a previous study reported a skin temperature reduction of 23 °C at the knee joint due to 20 min of focal knee joint cooling [18], assessment of thigh temperature (not on the intervention cite—knee joint) in our study was intended to see if temperature change in the quadriceps is involved in the cycling performance after joint cooling; if the cycling performance changes, it must be involved with quadriceps muscle function. Known effects of cold application include increased viscosity of synovial fluid [19], increased stiffness of mechanical properties in the ligamentous [20] and tendinous [21] structures, decreased threshold of alpha motor neuron recruitment [6], and decreased activation of inhibitory neurons [7]. Once our subjects experienced the aforementioned cooling effects, the set of cycling intervals after knee joint cooling resulted in a rapid increase in tissue temperature of the knee joint; thus, we assume that subjects may have benefitted from increased force production of knee extensors [22] and the angular velocity of the knee joint [5]. Additionally, knee joint cooling might have resulted in a deterioration of the negative feedback [23] (e.g., inhibiting α-motor neurons when fatigued). These neurological alterations could have made subjects pedal harder, which in turn leads to better cycling performance. A psychological benefit of having a better-perceived feeling during cycling due to a rapid warm-up could also be a good candidate to explain the results. As previously suggested [5], we believe that the neurophysiological mechanisms behind the observed cycling sprints and performance efficiency (e.g., fatigue perception and heart rate) are attributed to this combined effect of joint cooling and an explosive muscle contraction.

Muscle temperature significantly impacts athletic performance [24,25,26,27,28,29]; there is a general idea that the higher the muscle temperature, the better the force-generating capacity [24,30]. For example, a 1 °C difference in muscle temperature could affect up to 4% [25] or 10% [29] of athletic performance. In our study, the thigh temperatures were consistently increased when performing cycling sprints (during sets 1 through 5 regardless of condition: 32.8 °C, 33.0 °C, 33.4 °C, 33.7 °C, and 34.0 °C, respectively), and the cycling performance peaked at set 2 (Figure 3). Another important observation is that there was a thigh muscle temperature reduction due to knee joint cooling that had a positive impact on cycling performance (Figure 2 and Figure 3). There is a large body of data on declined muscle function with cold application to local muscle [31,32,33,34,35]; thus, muscle temperature reduction is associated with muscle function decline. Our data are an addition to the scientific evidence on this lack of connection between muscle temperature and muscle function [31], which may suggest that there could be a certain range of muscle temperature for an ideal muscle metabolism that produces maximum power output. Since core temperature might have a larger impact on muscle function than local muscle temperature [32], the relationship between muscle function and various locations of body temperature (e.g., core and joint temperature) should be examined in future studies.

There are several limitations and assumptions in our study. First, the generalization of knee joint cooling on cycling performance should be restricted to the specific situation where subjects performed five sets of cycling sprint intervals; thus, care should be taken when applying the results of our data to different types of cycling performance such as long-distance road cycling. Additionally, our results on a stationary ergometer cannot be directly applicable to road cycling because our testing protocol did not account for additional weights (e.g., a rider and a bike), road condition (e.g., slope and friction), and environmental condition (e.g., wind resistance, etc.). Neurological adaptation to temperature change (e.g., a set of cycling performances followed by knee joint cooling or vice versa) may vary between subjects due to physiological and psychological responses to a combined effect of cycling sprints and cold and/or personal experience of cold application and stationary cycling. Even within subjects, the results of our interventions could have been influenced by certain contributing factors such as lab conditions (e.g., ambient temperature and relative humidity), nutritional and hydration status, and maximal effort. Although heart rate and thigh temperature were collected simultaneously (the same sampling frequency), the recorded values of two variables at the same time points might have not been correspondent due to the heart rate lag [36]. Finally, we do not know how the observed results of cycling performance corresponded to changes in knee joint temperature. While the knee joint temperature was one of the primary contributing factors, attachment of the temperature probe to the knee joint was uncomfortable when pedaling at maximal effort. Future studies should attempt to record knee joint temperatures and to examine an interaction effect between the cycling performance and knee joint temperature.

## 5. Conclusions

A 20 min bilateral joint cooling with or without pre-cooling warm-up prior to cycling protocols seems to improve overall cycling performance during five sets of cycling interval protocols, as compared to the non-cooling condition. When compared to the non-cooling condition, the conditions with knee joint cooling showed four to five higher wheel revolutions with 19% and 2% less fatigue perception and heart rate, respectively. The direction of the results in each dependent variable clearly demonstrates an advantage of focal knee joint cooling on cycling sprint performance. We recommend coaches and athletes consider tying knee joint cooling on their training regimens.

## Figures and Tables

**Table 1:** Changes in cycling sprint performance.

		Set 1	Set 2	Set 3	Set 4	Set 5
Anaerobic Capacity (watt)	Condition 1	399 ± 41	407 ± 43	394 ± 37	385 ± 38	383 ± 38
		437 ± 33	436 ± 37	441 ± 38	434 ± 41	426 ± 42
	Condition 2	399 ± 41	407 ± 43	394 ± 37	385 ± 38	383 ± 38
		440 ± 46	455 ± 45	453 ± 40	439 ± 43	435 ± 44
	Condition 3	381 ± 39	385 ± 43	369 ± 39	354 ± 40	354 ± 41
		440 ± 43	445 ± 47	424 ± 44	405 ± 45	395 ± 44
Cadence (rpm)	Condition 1	149 ± 5	148 ± 8	150 ± 7	147 ± 7	147 ± 7
		165 ± 6	167 ± 7	165 ± 8	163 ± 8	161 ± 8
	Condition 2	147 ± 8	150 ± 8	147 ± 7	145 ± 9	144 ± 8
		160 ± 8	165 ± 7	164 ± 7	161 ± 8	159 ± 8
	Condition 3	144 ± 6	146 ± 7	145 ± 6	139 ± 7	140 ± 7
		163 ± 6	168 ± 6	163 ± 6	157 ± 7	155 ± 7

Values are mean ±95% confidence intervals. The average and peak values are reported at top and bottom, respectively. rpm: revolution per minute.

**Table 2:** Change in fatigue perception and heart rate.

		Baseline	Set 1	Set 2	Set 3	Set 4	Set 5
Fatigue perception (cm)	Condition 1	2.7 ± 0.9	2.5 ± 0.8	3.0 ± 0.8	3.6 ± 0.8	4.4 ± 0.9	5.3 ± 1.1
	Condition 2	2.7 ± 1.0	2.6 ± 0.9	3.0 ± 0.8	3.6 ± 0.9	4.2 ± 1.0	5.5 ± 0.9
	Condition 3	3.2 ± 1.0	3.1 ± 1.0	3.7 ± 1.1	4.5 ± 1.1	5.4 ± 1.2	6.5 ± 1.2
Heart rate (bpm)	Condition 1	73 ± 5	138 ± 7	143 ± 7	149 ± 7	152 ± 7	155 ± 7
	Condition 2	76 ± 7	135 ± 9	143 ± 7	149 ± 7	153 ± 6	157 ± 6
	Condition 3	79 ± 4	138 ± 7	147 ± 6	153 ± 6	155 ± 6	156 ± 6

Values are mean ±95% confidence intervals.

## Data Availability

Data will be shared upon acceptance of the manuscript.
